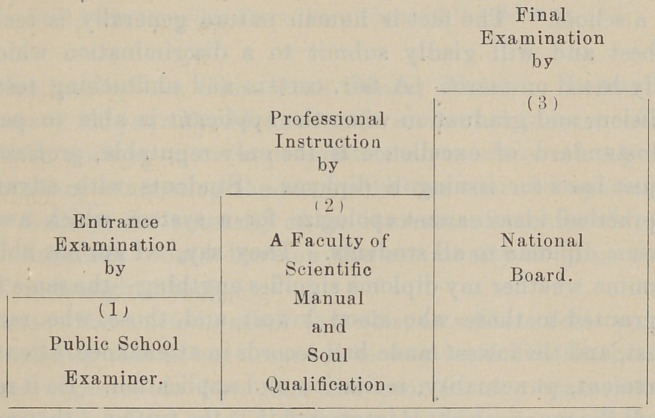# Four Requisites in Development

**Published:** 1896-01

**Authors:** M. F. Ault

**Affiliations:** Kokomo, Ind.


### Four Requisites in Development.

BY. M. F. AULT, M.D., D.D.S , KOKOMO, IND.

Read before the Tri-State Meeting in Detroit.

*Continued from Dec. No. Page* 577.

Specific means definite, particular, distinctive, special. Scientific pertains to accumulated, systematized, established knowledge and in our teaching refer to physiology, anatomy, chemistry, etc.

To illustrate the topic of specific scientific instruction we will use the first branch named—Physiology. We are somewhat surprised when the laity asks the necessity of giving physiology a place in the dental curriculum or as is more frequently heard—I don't see what that has to do with dentistry, but we are much more surprised when senior students are clamoring for something practical and asking why they must study this subject while to extract, restore and substitute teeth will require their constant attention. Can our college authorities satisfactorily answer this question? And also dispense with that languid, have-to-kill-time air of so many when in the scientific atmosphere of the college? We think the power which enforces scientific investigation can be vindicated, and if not the school must be one of manual training. Physiology, we will divide into two parts.—

1. Structural—Histological.
2. Functional—Vital.

And in the following synopsis we will exhibit the scope of physiological study for a dental student.

Physiology of the teeth
- 1 Structure
  - 1 Physical
    - 1 Hard
      - 1 Enamel
      - 2 Dentine
      - 3 Cementum
    - 2 Soft
      - 1 Pulp
      - 2 Peridental Mem.
  - 2 Chemical
- 2 Nutrition
  - 1 Proximate elements
  - 2 Digestion
  - 3 Secretion
  - 4 Absorption
  - 5 Circulation
  - 6 Respiration
  - 7 Animal heat
  - 8 Excretion
- 3 Relations
  - 1 Vascular
  - 2 Nervous
- 4 Functions
  - 1 Immediate
  - 2 Remote
- 5 Anæsthesia
  - 1 By internal agency
  - 2 By external agency
    - 1 Medicinal
    - 2 Hypnotic
- 6 Reproduction

It is not the purpose of the writer to give a lesson in histology or physiology, but to demonstrate his method of teaching. Notwithstanding the desire that I always feel to have my pupils appreciate this branch in all bearings my sole study is to emphasize its relation to their calling, hoping that this would prove an incentive to general investigation.

To state to a class that white fibrous connective tissue, nerve tissue, blood, blood-vessels, lymphatics, unstripped muscular tissue and cells are to be subjects for study awakes but little interest and inspires fear, but first to illustrate by a large figure that can be seen from all points in the room, showing care and skill in using the crayon, that a tooth is suspended in its socket by fibres of white connective tissue; that immediately surrounding the cementum is a very peculiar form of lymphatic—that about half way between the cementum and alveolar wall is a zone of nerves and blood-vessels and from the blood-vessels a spherical, nucleated cell works its way and by its differentiation it may become a builder of cementum a fibroblast—a bone-forming cell or an osteoclast.

The learner is at once intensely interested and confidence established, not only in the teacher, but in his own capacity for receiving and assimilating the subject. He is interested because he sees from the drawing that the explanation refers to vital structures within the field of his future operation and confidence to himself is secured by the large diagram fixing in his mind the points of size, shape, color, direction, location, relation, name and everything so suggestive as to almost reveal the function of each element. It is important to have the pupils follow in preparing the illustration as they will make a more minute inspection of the teacher's work—they have a chance to exhibit their personality in the work—to learn by doing, *i. e.*, to learn naturally and not have to go through the laborious, unsatisfactory and injurious struggle of committing to memory, besides there is a manual training in it and the faculty of form which is used in practical work is strengthened. Every one who proposes to instruct should make himself a black-board artist. Is the pupil's knowledge which is gained from the use of the microscope any more accurate than that gained by chalk diagrams? I think but very little. One reveals the object over-size—so does the other; one shows the location, shape and direction and this is true of the figure. I am not aiming to commend the one at the expense of the other but I am aiming to strengthen the black-board work by comparison. I believe the efforts of the learner in following the skillful crayon efforts of the professor is the proper introductory work to the use of the microscope as well as to make it an essential introductory for a lecture—the lens should supplement the deficiency of the best black-board illustration.

SECOND REQUISITE IN PROFESSIONAL CHARACTER.

Specific manual instruction.

This pertains to the chemical department of the college. Here the student is brought in contact with the text that breathes, feels pain, passes judgment and returns her verdict, here the student stands in doubt and realizes how weak he is. He is just beginning to appreciate what dentistry means and sees that no man can understand a doctrine until he applies it to his own life. He is engaging in something which tests his nerve, sympathy, integrity and his judgment. He feels that while there is pleasure in oral and written study that if he experiences the highest pleasure, he must express himself in a perfect object, and to illustrate his work he must construct—he must build something.

My purpose is to present the systems of manual training, and show how they may be employed to keep all students systematically employed.

1. The learner must make something useful from the first.
2. The learner must proceed by two steps.

(*a.*) A drawing which represents principles of construction.

(*b.*) Construction of the object by fullfilling the conditions of the drawing.

(*c.*) Specific soul instruction.

This pertains to the spirit which pervades the labor of life, and no student should be admitted into the sacred precints of the dental profession until his soul is in harmony with that which has blessed us with our present advancement and still leads us toward perfection.

We believe that the sphere of our efforts in its truest sense is a part of the eternal realm of truth. We believe that the strength and firmness of dentistry lies in our unity and unbounded reverential devotion to it. We believe that we must manufacture a true sentiment as a portion of posterity's legacy, and also that there is a spirit with which we must become imbued, and in the presence of the most high swear to make the development of the power of our profession our prime aim in life.

THE FOURTH REQUISITE.

The fourth requisite in professional development is sufficient strength of character in some colleges to pass through a needed transition. I think there is a call coming from this age and some college must respond. The college which is willing to submit the entrance qualification to a disinterested examiner from the public schools, to have its course of instructions strong enough to be able to submit the question of graduation to a National board whose sole purpose would be to replenish the profession with true ability is the college which will demonstrate the needed transition and in the most significient manner answer the call of the age. This would, in the truest sense, be a National Dental College; after a conservative survey of the situation, I would have all future aspirants enter the profession by three steps. I illustrate in this manner:

| Entrance Examination by | Professional Instruction by | Final Examination by |
|---|---|---|
| (1) | (2) | (3) |
| Public School Examiner. | A Faculty of Scientific Manual and Soul Qualification. | National Board. |

INFLTENCE ON STUDENTS.

Let us next examine the feasibility of this plan. According to the first step required no holder of a medical diploma or certificate from another dental college not of equal requirement would be exempt from examination, then all applicants must pass the literary test in the common English branches before a disinterested examiner, except holders of two-years teachers' license, or diploma from a high school, college or university. This requirement would rule out at least twenty-five percent of the candidates for admission. Is there a reason for continuing in the popular way —that inviting way which is practiced by colleges which talk much about repute? Let every one who has addressed a class containing twenty-five percent of illiterates—every teacher who has instructed the papers of unqualified students—every secretary who has gone through the disgraceful sham of entrance examination—every professor who for his head's sake, for his purse's sake or because of his own stupidity sanctioned the graduation of many incompetent, men answer whether there is any reason for transition and the best means of securing absolute silence with reference to the phrase, "Good Repute."

Some may say, where would you get your students? I have, during the preparation of this paper, interrogated several students and their countenances invariably bore a smile of approval—their utterances were "that is right;" "I would like a diploma from such a school." The fact is human nature generally is seeking the best and will gladly submit to a discrimination which is clearly based on merit. A fair, certain and unflinching test for admission, and graduation when the applicant is able to pass a fixed standard of excellence is the only reputable, professional and just basis for issuing a diploma. Students with advanced and practical ideas cannot apologize for a system which awards the same diploma to all students. They say, "I am not able to determine whether my diploma signifies anything—the same form was granted to those who stood lowest and those who ranked highest, and the lowest made bad records in attendance, attention, deportment, punctuality, recitation and application. So it seems that all the management is interested in is the tuition—they admit us for money and graduate us to get rid of us." It is evident that every student expects to pass and it is felt to a certainty that the faculty cannot refuse to graduates, unless there is a very unusual violation of rules. When we first read the catalogue we thought a standard in literary training had been adopted and that students measuring up to certain percent would be admitted, but we were much surprised to find no examination or what there was, an object subterfuge, the meanest evasion, a forlorn artifice.

We all attend the same time—three years. Some are faithful and able to master the course in half of three years, others are stupid and indifferent and need three years and probably five; others are a drunken, beastly set and could not pass short of ten years if application to a problem which must be solved is the right requirement. We are certain that if a pupil could be eligible to an examination before a national committee upon recommendation of the faculty that it would be justice to those who work hard and feel the need of saving time and money. Also it would prove the greatest incentive to the mediocre and a fair warning to those who are willfully stupid in brain, hand, profession or spirit.

[*To be Continued.*]

## Figures and Tables

**Figure f1:**
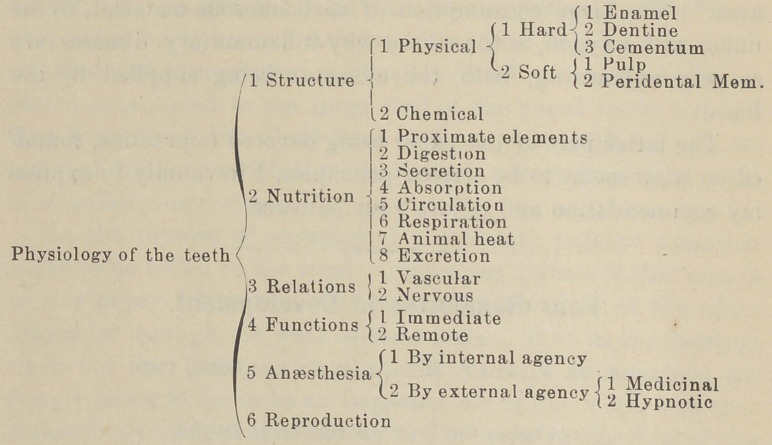


**Figure f2:**